# Assessment methods in laparoscopic colorectal surgery: a systematic review of available instruments

**DOI:** 10.1007/s00384-023-04395-9

**Published:** 2023-04-19

**Authors:** Tom van Zwieten, Sietske Okkema, Marc van Det, Ilona Pereboom, Nic Veeger, Jean-Pierre Pierie

**Affiliations:** 1 Department of Surgery, Medical Centre Leeuwarden, Henri Dunantweg 2, 8934AD Leeuwarden, The Netherlands; 2 Department of Surgery, Hospital Group Twente, Almelo, The Netherlands; 3 Postgraduate School of Medicine, University Medical Centre Groningen (UMCG), Groningen, The Netherlands; 4 Department of Epidemiology, University of Groningen, University Medical Centre Groningen, Groningen, The Netherlands

**Keywords:** Laparoscopic colorectal surgery, Laparoscopic training, Procedure-based assessment, Summative assessment

## Abstract

**Background:**

Laparoscopic surgery has become the golden standard for many procedures, requiring new skills and training methods. The aim of this review is to appraise literature on assessment methods for laparoscopic colorectal procedures and quantify these methods for implementation in surgical training.

**Materials and methods:**

PubMed, Embase and Cochrane Central Register of Controlled Trials databases were searched in October 2022 for studies reporting learning and assessment methods for laparoscopic colorectal surgery. Quality was scored using the Downs and Black checklist. Included articles were categorized in procedure-based assessment methods and non-procedure-based assessment methods. A second distinction was made between capability for formative and/or summative assessment.

**Results:**

In this systematic review, nineteen studies were included. These studies showed large heterogeneity despite categorization. Median quality score was 15 (range 0–26). Fourteen studies were categorized as procedure-based assessment methods (PBA), and five as non-procedure-based assessment methods. Three studies were applicable for summative assessment.

**Conclusions:**

The results show a considerable diversity in assessment methods with varying quality and suitability. To prevent a sprawl of assessment methods, we argue for selection and development of available high-quality assessment methods. A procedure-based structure combined with an objective assessment scale and possibility for summative assessment should be cornerstones.

**Supplementary Information:**

The online version contains supplementary material available at 10.1007/s00384-023-04395-9.

## Background

Traditionally, surgical procedure training is done according to the master-apprentice model. Surgical residents are assessed in the operating room through supervision and formative feedback [1]. This type of feedback is highly subjective and often undocumented. Therefore, it is not transferrable between different supervisors (i.e. masters) and different teaching hospitals. In order to structure surgical training and assessment, training programs have broadly adopted General Rating Scales (GRSs) such as the Objective Structural Assessment of Technical Skills (OSATS) and Global Operative Assessment of Laparoscopic Surgery (GOALS) [2, 3]. Laparoscopic surgery is considered markedly different from open surgical procedures, with specific technical aspects and different learning curves, and therefore requires more appropriate assessment methods [4, 5]. For this purpose, specific GRSs for laparoscopic surgery, such as the GOALS, have been developed.

Although GRSs were developed and implemented in surgical training worldwide to give more objectivity to the assessment of competency in the traditional ‘master-apprentice model’, they are not undisputed. A drawback of GRSs is that they only provide general information for formative assessment on residents’ surgical technique and lack feedback for specific steps of a surgical procedure. For this reason, the GRSs’ capability to objectively evaluate the quality of a specific surgical procedure can be questioned and they may be considered unsuitable for high-stake examinations (i.e. summative assessment) [6]. Furthermore, the validity of the GRSs is debated in literature because of their inability to differentiate between performance rates at similar experience levels and because of a lack of procedure-specific features [7, 8].

The individual judgement of the supervising surgeon, supported by general rating scales, is probably no longer sufficient. An objective, clearly defined, procedure-based assessment of operative performance is thought to be important as a basis for constructive feedback during new procedures. Also, the rate of advancement of technical skills in surgical trainees can be monitored more adequately with rotating supervisors.

Laparoscopic colorectal surgery (LCS) is considered advanced minimally invasive surgery with a long and variable learning curve. Some studies describe an increased rate of conversion and complications during the learning period [9, 10]. Efforts have been made to develop assessment methods specifically for LCS to objectify progression in operative performance, both technically and non-technically. An objective assessment allows monitoring of the learning curve and provides a basis for constructive, structured feedback. Additionally, objective assessment methods could be helpful in credentialing new procedures.

Although the OSATS is generally accepted and applied worldwide for assessing surgical procedures, there are no commonly used assessment methods for the evaluation of laparoscopic colorectal surgery. Haug et al. published a scoping review in December 2021 with an overview of available tools for laparoscopic colorectal surgery [11]. Their primary focus was to assess validity of available skill assessment methods in laparoscopic colon surgery. Only a few tools present substantial validity for development and use.

The primary goal of this systematic review is to provide an overview of available assessment methods other than GRSs for laparoscopic colorectal surgery and consider possibilities for summative assessment and, ultimately, credentialing. Furthermore, we evaluated whether assessment methods for laparoscopic colorectal surgery are described adequate and transferable. The results could provide a basis for further research into the development and optimization of high-quality assessment methods for laparoscopic colorectal surgery.

## Materials and methods

The PRISMA (Preferred Reporting Items for Systematic Reviews and Meta-analyses) statement was used as a guide for this systematic review [12] (see supplementary files).

### Search protocol

A systematic search was performed to identify eligible studies by searching three electronic databases: PubMed, Embase and the Cochrane Central Register of Controlled Trials. The search was focused on learning and assessing laparoscopic colorectal surgery. References of the included articles were screened for additional studies which might have been missed in the search. The search strategy was published on https://searchrxiv.org/ (https://doi.org/10.1079/searchRxiv.2022.00067).

The PubMed search included the following search terms and this syntax was adapted for the other databases: (“Laparoscopy “[Mesh] OR laparoscop*[tiab] OR minimally invasive surg*[tiab] OR minimal invasive surg*[tiab]) AND (“Intestine, Large”[Mesh] OR “Colorectal Surgery”[Mesh] OR large intestine*[tiab] OR colorectal*[tiab] OR colon*[tiab] OR rectum[tiab] OR rectal[tiab] OR cecum[tiab] OR hemicolectom*[tiab] OR colectom*[tiab] OR sigmoid*[tiab] OR abdominoperineal resection*[tiab] OR abdomino perineal resection*[tiab] OR abdominoperineal excision*[tiab] OR abdomino perineal excision*[tiab] OR Low anterior resection*[tiab]) AND (“Learning”[Mesh] OR “Education”[Mesh] OR “education”[Subheading] OR “Clinical Competence”[Mesh] OR “Video Recording”[Mesh] OR learn*[tiab] OR education[tiab] OR competenc*[tiab] OR training*[tiab] OR trainee*[tiab] OR video[tiab] OR videos[tiab] OR skill*[tiab] OR global rating scale*[tiab] OR OSATS[tiab] OR assessment*[tiab] OR key step*[tiab]) NOT (animals[mesh] NOT humans[mesh]).

### Study selection

The final search was performed on October 5, 2022. All hits were selected and checked for duplicates. The two assessors, TvZ and SO, independently selected potential eligible studies by title and abstract. There were no limits for publication year. Inclusion criteria were that the articles are written in English and report on assessment methods for laparoscopic colorectal surgery. The full article was read when information in the title and abstract met the inclusion criteria (Table 1). Articles reporting about robotic colorectal surgery, open colorectal surgery and trans anal total mesorectal excision (TaTME) were excluded (Table 1). The eligibility of articles was then evaluated by both assessors independently. Articles were included when consensus was reached. Disagreement between the two assessors was resolved by discussion with a third and fourth reviewer (NV and JP), after which consensus was reached.

**Table 1:** Inclusion criteria

Inclusion criteria	Exclusion criteria
-Assessment methods for laparoscopic colorectal surgery	-Robotic colorectal surgery
-English written	-Open colorectal surgery
	-Assessment of non-technical skills only
	-Assessment of medical students
	-Transanal total mesorectal excision (TaTME)
	-Reviews, conference abstracts and editorials

### Quality assessment

Quality assessment was performed by TvZ, and possible concerns were discussed with JP and NV. The tool used for quality assessment was the checklist of Downs and Black [13]. The Downs and Black checklist is divided into 4 subscales after power is excluded as a subscale. The checklist provides a numeric score, ranging from 0 to 27. According to an evaluation by Deeks et al., this is one of the most adequate tools to assess methodological quality and the risk for bias, of randomized, non-randomized and observational studies [14].

### Categorization

In order to get an overview of a potentially heterogeneous selection of developed assessment methods and what they can be used for, studies were categorized. Assessment methods were discriminated based on whether they were developed as procedure-based assessment method or not. A second distinction was made between capability for formative assessment (feedback) or high-stake decision making (summative assessment).

## Results

### Study selection

**Fig. 1:**
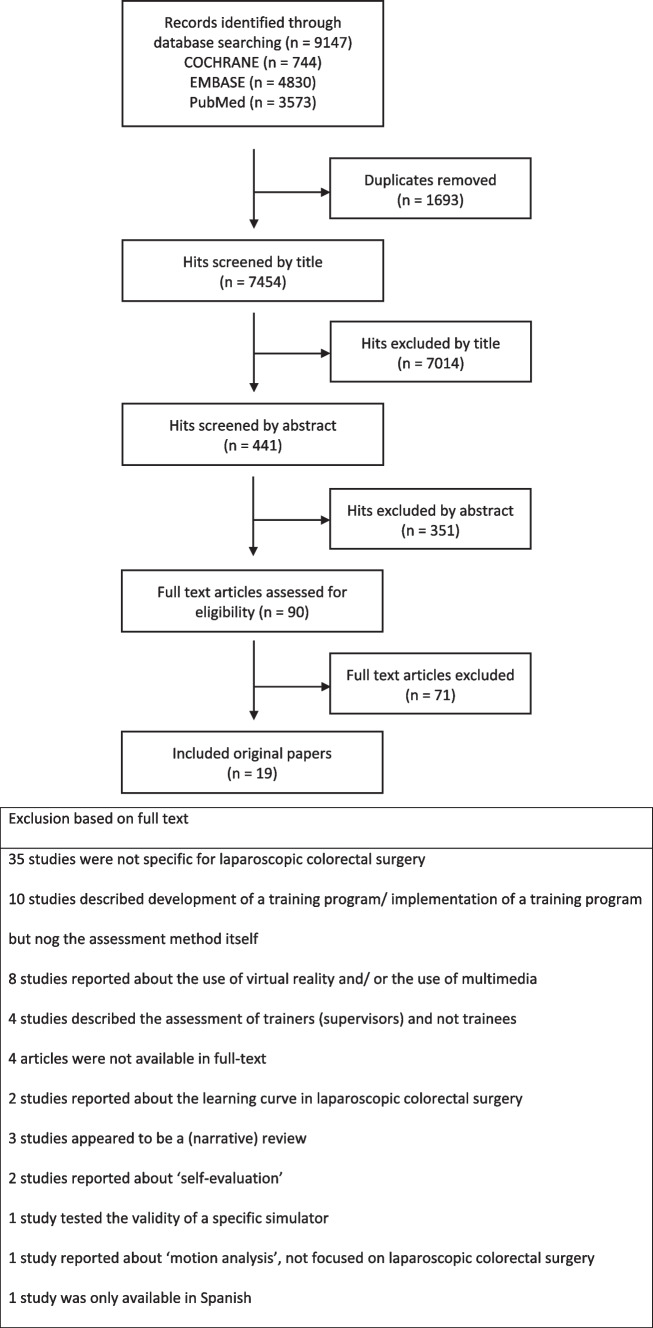
Prisma flowchart of systematic review of literature

The study selection process is described in the PRISMA flowchart (Fig. 1). A total of 9147 citations (3573 PubMed, 4830 Embase and 744 Cochrane Library) were identified by our search strategy. About 1693 duplicates were removed. After screening title and abstract, 90 articles were retrieved for detailed evaluation. Based on the predefined selection criteria (see Table 1), 24 articles were included. Five studies were subsequently excluded after full-text evaluation and discussion with a third and fourth reviewer because they did not meet the inclusion criteria. Three of these articles were not specific to colorectal surgery, one article appeared to be a review, and one article was not available in English. Additional screening of the references of the selected articles did not generate any additional articles. After consensus was reached, a total of 19 studies were included. All studies were observational studies. There were no randomized controlled trials that met the inclusion criteria.

### Study quality

The median quality score according to the Downs and Black checklist was 15 (range 0–26) for the non-randomized trials (Table 2). Overall, most publications were of average quality and lacking a good score in at least two out of four subscales (reporting, internal validity, external validity and confounding). However, two research groups with respectively 6 (Table 2: nos. 3, 9–13) and 2 (Table 2: nos. 16, 17) publications had high quality scores. Eleven studies reached the maximum score on external validity. Not a single study reached the maximum score for reporting or for internal validity or confounding. Two studies reached 9 out of 10 points in reporting (Table 2: nos. 8, 17). The high scores on study quality were predominantly reached in the studies that used the Delphi methodology.

**Table 2:** Inclusion criteria

Exclusion based on full text
35 studies were not specific for laparoscopic colorectal surgery
10 studies described development of a training program/implementation of a training program but not the assessment method itself
8 studies reported on the use of virtual reality and/or the use of multimedia
4 studies described the assessment of trainers (supervisors) and not trainees
4 articles were not available in full text
2 studies reported about the learning curve in laparoscopic colorectal surgery
3 studies appeared to be a (narrative) review
2 studies reported about ‘self-evaluation’
1 study tested the validity of a specific simulator
1 study reported about ‘motion analysis’, not focused on laparoscopic colorectal surgery
1 study was only available in Spanish

### Study characteristics

A large heterogeneity of studies was found despite categorization using different methods to develop an assessment method. Most of the included studies were categorized as procedure-based assessment methods and only a small number appeared applicable for summative assessment. A few studies stated to have the potential to be used for summative assessment.

#### Procedure-based assessment

Fourteen studies were categorized as (part of) a newly developed PBA for colorectal procedures [15–24].

Dath et al. created two procedure-specific assessment methods for the Nissen fundoplication and low anterior resection [15]. In their study, these assessment methods are called ‘operative component rating scales (OCRS)’. The OCRS were developed by identifying procedural components by surgical experts. Surgical residents were assessed on the basis of a video recording of an operation performed on a pig model with the OCRS and a global rating scale. The assessment technique showed reasonable reliability. The authors state that the technique has the potential for credentialing surgeons in laparoscopic procedures (i.e. summative assessment).

Sarker et al. developed a list of key- and subtasks for three laparoscopic colorectal procedures: right hemicolectomy, sigmoid colectomy and anterior resection [19, 25]. Tasks were identified by hierarchical task analysis and by linking these tasks to a Likert scale (global scoring system). Live operations of both consultant surgeons and surgical trainees were assessed. Good reliability was found for both generic and specific technical skills. Also, construct validity for the Likert scoring of generic and specific technical skills was significant.

Palter et al. and Dijkstra et al. used the Delphi methodology to identify key- and substeps of a right colectomy and sigmoid colectomy in order to develop an evaluation tool [16, 17]. Both state that the developed steps could be used for a procedure-specific evaluation tool as well as for credentialing practicing surgeons. Palter et al. hypothesize that their stepwise evaluation tool could facilitate the training of surgical residents and potentially shorten learning curves. The long-term objective was to create a validated evaluation tool for use in residency training programs and for credentialing surgeons. In 2012, Palter and Grantcharov validated the procedure-specific evaluation tool for laparoscopic right hemicolectomy and laparoscopic sigmoid colectomy and demonstrated acceptable interrater reliability and construct validity in a follow-up study [26]. Study participants were general surgical staff and surgical residents.

Assessment of technical skills in laparoscopic colorectal surgery has been applied in the ‘Lapco’ national training program in the UK [27]. Miskovic et al. developed a monitoring tool for training progression in laparoscopic colorectal surgery by creating a generic task analysis and scoring system for laparoscopic colorectal resections with the use of scientific literature, internet sources, educational videos and books [20]. The final version was approved by the educational committee of the National Training Program, represented by 12 expert laparoscopic surgeons from different hospitals and an educationalist. This resulted in a list with four generic task zones and twelve generic task steps for laparoscopic colorectal resections, combined with a scoring system. The monitoring tool was found to be highly practical, valid and reliable.

To implement competency assessment in laparoscopic colorectal surgery, Miskovic et al. investigated the potential of observational clinical human-reliability analysis (OCHRA), for competency assessment at a specialist level in terms of construct and concurrent validity [28]. OCHRA is a structured approach to the detection of errors and near-misses during surgery, which is implemented through the analysis of OR video recordings [29, 30]. A task analysis was performed using this approach in accordance with a previously described monitoring tool [20]. The procedure showed construct validity and concurrent validity, and the authors concluded that OCHRA is a valid method to assess surgical performance at a specialist level with the potential to be used for recertification.

Miskovic et al. developed and validated a competency assessment tool (CAT) for technical performance in laparoscopic colorectal surgery at a specialist level for high-stake assessment i.e. summative assessment [31]. This resulted in a form with 4 columns representing task components and 4 domains representing generic skills. A descriptive scoring system was used. The CAT was validated, and the authors stated that the CAT could be used for summative assessment for technical competency in laparoscopic colorectal surgery at a specialist level.

In 2015, Mackenzie et al. validated the CAT in a clinical setting. This study showed that the CAT is able to distinguish the level of competency of consultant surgeons [21]. Also, the study showed that technical competency was dependent on supervised training volume and not on the overall laparoscopic colorectal volume, consistent with the theory of deliberate practice.

Haug et al. 2022 [24] developed a procedure-specific tool for skill assessment for left- and right-sided laparoscopic complete mesocolic excision, using the Delphi methodology. The study provides evidence of content validity and is proposed to be useable for future assessment and certification of surgeons performing laparoscopic complete mesocolic excision.

Ni et al. [32] performed an error analysis on data from the UK’s National Training Programme. The study identified operative errors that were more likely to lead to a negative competency assessment, thereby suggesting critical determinants of surgical competency [30]. Knowledge of these determinants could help tailor surgical training modules.

Champagne et al. modified existing assessment scales to produce a tool for assessment of right-sided laparoscopic hemicolectomy [33]. The result was the ASCRS (American Society of Colon and Rectal Surgeons) tool to evaluate performance on both specific and more general metrics in laparoscopic colorectal surgery. The tool was validated and showed good interrater reliability in a large group with blinded, trained experts. It was also able to differentiate between competency levels using stepwise progression based on level of experience and could therefore potentially be used for summative assessment.

Nakayama et al. used the Delphi method to develop an assessment tool for a laparoscopic sigmoidectomy [22]. Although the study refers to previous assessment methods, they consider these methods unsuitable for Japanese practice due to differences in surgical procedure. The novel scale may be applicable for evaluating trainees in East Asian countries.

In 2020, Curtis et al. found that surgical skills can be objectively and reliably measured in complex cancer interventions [23]. They reached this conclusion after they developed an assessment tool for laparoscopic TME was developed using the Delphi methodology. Surgical skills were measured using this tool and were reported along with surgical outcomes in procedures performed by consultant experts. Variation in technical performance was associated with both clinical and pathological outcomes.

#### Non-procedure-based assessment

Five studies were categorized as “non-procedure-based assessment”.

Glarner et al. created a tool to assess performance in multiple areas (covering technical as well as non-technical skills) in laparoscopic segmental colectomies [18]. A domain expert identified key components of laparoscopic segmental colectomies and coupled these components to a rating scale expressed in the amount of assistance required by the resident. General technical skills of surgical residents were assessed with a procedure-specific assessment and OSATS, and the evaluation of non-technical skills was assessed with a variation on existing assessment methods for NOTECHS. The result was an assessment tool that incorporated three domains of resident performance in one; procedure-specific general technical performance and non-technical performance. The assessment tool was validated and concluded to be useful for direct, structured feedback and assessment in laparoscopic segmental colectomies.

A multi-modality training was evaluated by Jenkins et al. [34]. The training consists of various aspects of preparation, technical skill and postoperative care. Operations were divided into modules varying in difficulty. The study found the multi-modal training to be a performance-enhancing intervention in laparoscopic fellows, which leads to rapid proficiency gain in laparoscopic colorectal surgery without an increase in major morbidity.

Alba Mesa et al. used the failure mode and effect analysis (FMEA) in laparoscopic surgery training. This is a standardized approach to assess a complex process, identifying elements carrying high risks [35]. The method is derived from industries outside of healthcare, such as the aerospace industry, but has also been tested in various clinical settings. A FMEA matrix was developed for laparoscopic sigmoidectomy training using pig models by an interdisciplinary working group participating in each step of the operation. The authors conclude that the implementation of FMEA can contribute to a reduction of the risk of human error and improve patient safety during training.

Foster et al. evaluated the validity and reliability of OCHRA for evaluation of technical performance of laparoscopic rectal surgery [36]. A hierarchical task analysis was created, and potential errors were defined. OCHRA was used to identify technical errors in videos of laparoscopic rectal surgery performed by consultant surgeons. The videos were assessed by the laparoscopic fellow surgeon. OCHRA appeared to be a valid and reliable method for evaluating technical performance in laparoscopic rectal surgery.

Ichikawa et al. evaluated a mentor-tutoring system in a general hospital setting. The study evaluated the learning curve of a trainee in the author’s centre [37]. The system consisted of intensive training sessions and sharing of expert knowledge. A similar learning curve was found when compared to high-volume centres for achieving competency. Weekly mentor tutoring was suggested to be an effective method for providing laparoscopic colorectal surgery training in a general hospital.

### Formative and summative assessment

Summative assessment methods can be used for high-stake examinations i.e. deciding whether a participant is capable of performing a surgical procedure. Assessment methods can also be developed as a tool for structured feedback during the learning curve: formative feedback. Beside the aforementioned distinction between PBA and non-PBA, assessment methods can also be divided into formative or summative depending on their current or future perspective to be used as such. An overview is given in Table 3. Only three studies were suitable for summative assessment [21, 28, 31]. Five studies had future potential for summative assessment [15–17, 24, 33]. Most of the assessment methods which were proposed as summative also have the capability to be used for formative feedback. Regardless of the parameters being measured, assessment methods with potential for summative assessment have some sort of cut-off score where a certain threshold has to be reached for credentialing Table 4.

**Table 3:** Quality assessment according to the Downs and Black checklist [12]

	Article	Reporting (0–10)	External validity (0–3)	Internal validity — bias (0–7)	Internal validity—–confounding (0–6)	Total (0–26)
1	Alba Mesa et al. [35]	6	2	2	0	10
2	Champagne et al. [33]	6	3	5	2	16
3	Curtis et al. [23]	8	3	5	2	18
4	Dath et al. [15]	7	3	2	2	14
5	Dijkstra et al. [17]	7	3	3	2	15
6	Foster et al. [36]	7	2	4	2	15
7	Glarner et al. [18]	6	2	2	3	13
8	Ichikawa et al. [37]	9	1	3	0	13
9	Haug et al. [24]	6	3	6	3	18
10	Jenkins et al. [34]	4	3	3	2	12
11	Mackenzie et al. [21]	7	3	4	4	18
12	Miskovic et al. [28]	7	3	6	4	20
13	Miskovic et al. [4]	6	3	6	3	18
14	Miskovic et al. [20]	8	2	4	3	17
15	Nakayama et al. [22]	6	3	4	2	15
16	Ni et al. [32]	4	3	4	2	13
17	Palter and Grantcharov [26]	7	3	4	5	19
18	Palter et al. [16]	9	3	4	4	20
19	Sarker et al. [19]	6	3	3	2	14

**Table 4:** Overview of applicability for summative and/or formative assessment

**Author**	**Formative**	**Summative**	**Note**
***Procedure-based assessment***			
Dath et al. [15]	X		Future possibilities for summative assessment
Sarker et al. [19]	X		
Palter and Grantcharov [26]	X		Future possibilities for summative assessment
Palter et al. [16]	X		
Dijkstra et al. [17]	X		Future possibilities for summative assessment
Miskovic et al. [20]	X		
Miskovic et al. [4]		X	
Miskovic et al. [28]		X	
Mackenzie et al. [21]		X	
Ni et al. [32]	-	-	Error identification for credentialing
Champagne et al. [33]	X		Future possibilities for summative assessment
Curtis et al. [23]		X	Not used for formative/summative assessment in this study
Nakayama et al. [22]	-	-	Development of steps for future assessment tool
Haug et al. [24]	X		Future possibilities for summative assessment
***Non-procedure-based assessment***			
Glarner et al. [18]	X		
Alba Mesa et al. [35]	X		
Jenkins et al. [34]	X		
Foster et al. [36]	X		
Ichikawa et al. [37]	X		

## Discussion

This systematic review identified and analysed 19 studies on assessment of surgical skills for laparoscopic colorectal surgery with large heterogeneity. Most assessment methods were developed as PBA, and only a few studies described tools that were applicable for summative assessment. The Delphi method was used often among studies with high methodological quality. Considering the large number of studies conducted to develop or validate assessment methods for laparoscopic colorectal surgery, we believe the next step is to decide which method should be used as an alternative for GRSs in assessing surgical competence regarding laparoscopic colorectal surgery on a resident and specialist level. After that, further development and optimization of the chosen assessment method should be the focus. A guideline for specific assessment methods, like the LAP-VEGaS video assessment tool is for videos of laparoscopic procedures, could be valuable in ensuring a certain quality for new developed assessment methods [38].

Residents or surgeons who are in their learning curve for laparoscopic colorectal surgery already possess general technical skills, and therefore mainly benefit from procedure-based feedback. Procedure-based assessment enables supervising surgeons to provide specific feedback that could facilitate improvement of a trainee’s performance of a procedure [39]. Furthermore, summative assessment methods are needed to ensure competent surgeons can be credentialed for specific procedures. Although several studies pose future perspectives for summative assessment, only four of the currently available assessment methods were suitable for summative assessment of laparoscopic colorectal surgery. When implementing a summative assessment, a cut-off score is required, which raises new questions. For example: should false-positive scores (falsely assessed as capable to perform a procedure independently) be accepted in determining the cut-off score? In an earlier publication, we chose for maximal specificity because an incorrect estimate of being capable of performing an operation independently was stated as undesirable [40].

Our review identified a large variety of different assessment methods developed for laparoscopic colorectal surgery. With regard to the quality of the studies, the studies that scored relatively high on quality assessment used the Delphi methodology to develop a procedure-based assessment method. This seems to be concordant with the findings of Haug et al., in which studies with high evidence of validity predominantly used the Delphi method [11]. Other assessment methods that were developed in a more ad hoc way, without a detailed description of the applied task analysis, were ranked lower regarding methodological quality.

A few of the described assessment methods are already incorporated in local or in national training programs. The National Training Program for laparoscopic colorectal surgery in the UK appears to be very innovative in its application of procedure-based assessment methods for summative assessment [20, 21, 24, 31, 41]. A competency assessment tool was developed and validated in clinical practice on a specialist level and appeared to be able to distinguish the competency level of consultant surgeons. Also, Mackenzie showed findings consistent with the theory of deliberate practice: expertise is not exclusively related to the volume of experience but to time spent practicing with constructive feedback [21, 42]. This endorses the importance of structured feedback in which procedure-based assessment methods can help.

## Conclusion

A large number of heterogeneous assessment methods for laparoscopic colorectal surgery are available in the literature. To prevent a sprawl of assessment methods, the authors suggest further development of existing procedure-based assessment methods, into a widely accepted and implemented tool which are also suitable for summative assessment. The use of the Delphi method results in high-quality procedure-based assessment methods reflected by its use in the high-quality articles. We encourage further development and optimization of the methods used in The National Training Program for laparoscopic colorectal surgery in the UK and promising methods such as those of Palter et al., Champagne et al. and Dijkstra et al. These studies have substantial overlap in the development of assessment method and the specific steps to assess. This suggests that there is already some agreement on the wish as well as the means to develop these assessment methods for laparoscopic colorectal surgery. The national boards of surgeons are encouraged to discuss preferences and to implement the suggested assessment methods in their training programs.

### Supplementary Information

Below is the link to the electronic supplementary material.

Supplementary file1 (DOCX 32 KB)
